# Phenotype Heterogeneity and the Association Between Visual Acuity and Outer Retinal Structure in a Cohort of Chinese X-Linked Juvenile Retinoschisis Patients

**DOI:** 10.3389/fgene.2022.832814

**Published:** 2022-03-04

**Authors:** Qingge Guo, Ya Li, Jiarui Li, Ya You, Changgeng Liu, Kang Chen, Shuyin Li, Bo Lei

**Affiliations:** ^1^ Henan Eye Institute, Henan Eye Hospital, Henan Provincial People's Hospital, Zhengzhou, China; ^2^ Henan Clinical Research Center for Ocular Diseases, People’s Hospital of Zhengzhou University, Zhengzhou, China; ^3^ School of Medicine, People’s Hospital of Henan University, Henan University, Zhengzhou, China

**Keywords:** X-linked juvenile retinoschisis, SS-OCT, AO, ERG, next-generation sequencing

## Abstract

**Purpose:** X-linked juvenile retinoschisis (XLRS), caused by mutations in the *RS*1 gene, is an X-linked recessive inherited disease that typically involves both eyes in the first 2 decades of life. Recently, the phenotype heterogeneity of this condition has drawn increasing attention. We reported various phenotypes caused by *RS1* gene mutations in eleven patients from ten Chinese families.

**Methods:** Data on the medical history of the patients from ten Han families of central China were collected. Ophthalmic examinations including best-corrected visual acuity (BCVA), fundus photography, ultra-wide-angle sweep source optical coherence tomography (SS-OCT), and electroretinography (ERG) were performed. Adaptive optics (AO) images were acquired to evaluate the cone photoreceptor mosaic when applicable. Venous blood of the probands and their family members was collected, and DNA was subjected to sequencing based on next-generation sequencing with a custom-designed targeted gene panel PS400 for inherited retinal diseases. Validation was performed by Sanger sequencing and cosegregation. Pathogenicity was determined in accordance with the American College of Medical Genetics and Genomics (ACMG) guidelines.

**Results:** Ten *RS1* mutations, including eight missense mutations and two terminator mutations, were identified in 10 XLRS families. c.657C > A (*p*.C219X) was a novel mutation in this cohort. These patients showed a variety of clinical phenotypes, including fovea schisis, bullous retinoschisis, and macular or peripheral atrophy. Fifteen eyes of eight patients exhibited macular retinoschisis, and twelve eyes of seven patients exhibited peripheral retinoschisis. In addition, three patients showed asymmetrical fundus manifestations. Of importance, three patients without macular retinoschisis were misdiagnosed until genetic testing results were obtained. AO showed a decrease in cone density and loss of regularity in the cystic schisis macular of XLRS. Furthermore, the BCVA was associated with the photoreceptor inner segment and outer segment (IS/OS) thickness.

**Conclusion:** With complicated clinical manifestations, a considerable portion of XLRS patients may present various phenotypes. It should be noted that asymmetry in fundus appearance in both eyes could lead to misdiagnosis easily. Thus, genetic testing is crucial for making a final diagnosis in those patients who are suspected of having amblyopia, bilateral or unilateral macular atrophy, or conditions presenting an asymmetric fundus appearance. In addition, the residual cone photoreceptor structure was critical for the maintenance of useful vision.

## Introduction

X-linked juvenile retinoschisis (XLRS) is a recessive inherited retinal disease characterized by mild-to-severe central vision loss, spoke-like schisis patterns in the fovea, splitting of the inner layers of the retina, and functionally a negative electroretinography (ERG) pattern caused by a significant decrease in the b-wave amplitude (Molday et al., 2012). The prevalence of XLRS is estimated to be 1:5000 to 1:20000. XLRS is caused by mutations in the *RS1* gene (NM_000330) on chromosome Xp22.1. RS1 encodes retinoschisin, a protein in the extracellular matrix that serves as an intercellular adhesive. Mutations in this gene can result in retinoschisis, *i.e.,* separation between retinal layers and formation of cystic cavities within the retina (Smith et al., 2020). With age, the cyst schisis may collapse, coalesce, or even lead atrophy gradually (Rao et al., 2018). It was reported that foveal involvement was observed in almost all XLRS patients, while peripheral retinoschisis accounted for 30–71%, and retinal detachment occurred in 3–16% of eyes. Patients with bullous retinoschisis in childhood tended to present with strabismus, nystagmus, vitreous hemorrhage or irregularly shaped pupil, and retinal detachment (Molday et al., 2012; De Silva et al., 2021). Various evident manifestations and complications often hamper physicians from considering the real etiology of this genetic condition, *RS1* mutations, and consequently lead to misdiagnosis.

XLRS patients usually exhibited similar morphological and functional changes bilaterally (Molday et al., 2012). However, the unilateral occurrence of complications such as vitreous hemorrhage and retinal detachment may cause remarkable differences in retinal anatomy and visual function between the two eyes. Furthermore, although asymmetric XLRS has rarely been reported, we did see an increasing number of such patients who were verified harboring *RS1* mutations, partially due to the application of advanced image systems which provided a much broader view and more detailed structure of the fundus. Here, we report eleven XLRS patients from ten Chinese families, some of whom had atypical fundus appearances such as asymmetric fundus or macular atrophy. Furthermore, we evaluated the correlations between the best-corrected visual acuity (BCVA) and the thickness of the retinal fovea, outer nuclear layer (ONL), and the photoreceptor inner segment and outer segment (IS/OS). Cone mosaic was also evaluated with adaptive optics (AO) when applicable.

## Methods

### Targeted Sequencing and Genetic Analysis

Eleven XLRS patients were diagnosed in ten Han families from central China. Venous blood was collected and subjected to next-generation sequencing with a custom-designed targeted gene panel (PS400) containing 376 genes associated with inherited retinal diseases (Fu et al., 2021; Zhang et al., 2021; Zhu et al., 2021). The enriched DNA libraries (150-200bp) were sequenced on an Illumina Nova Seq 6,000 sequencer (Illumina, San Diego, CA), and the average sequencing depth was 200 x. The raw reads were aligned to the human genome reference (hg19) using the BWA software (Burrows Wheeler Aligner, v0.7.12-r1039). PolyPhen-2 (Polymorphism Phenotyping v2), Mutation Taster, and SIFT (Sorting Intolerant From Tolerant) were used to assess the possible pathogenicity of these mutations (Fu et al., 2021; Zhang L. et al., 2021; Zhu et al., 2021). Validation was performed by Sanger sequencing, and pathogenicity analysis was performed in accordance with the American College of Medical Genetics and Genomics (ACMG) guidelines (Richards et al., 2015).

### Clinical Assessment

Ophthalmic examinations include the BCVA, slit lamp microscope, fundus photography, and swept source optical coherence tomography (SS-OCT, VG200D, SVision Imaging, Henan, China). AO images (rtx1, Imagine Eyes, Orsay, France) were acquired when applicable. Full-field electroretinography was recorded following the International Society for Clinical Electrophysiology of Vision (ISCEV) standard (Robson et al., 2018).

To evaluate the association between visual function and the retinal structure, XLRS patients were divided into two groups according to the severity of visual impairment. In group A, the BCVA was greater than or equal to 0.1. Patients in group B had lower BCVA. Visual acuity less than 0.01 was counted as 0.001. The retinal fovea thickness, outer nuclear layer (ONL) thickness, the photoreceptor inner segment and outer segment (IS/OS) thickness were detected (Makiyama et al., 2013). The distribution property of the data was analyzed. Data were analyzed by the unpaired T test, since normal distribution was confirmed. In addition, the associations between the BCVA and the thinkness of the fovea, ONL, and IS/OS were tested with linear regression. *P*<0.05 indicated statistical significance.

All procedures were performed according to the tenets of the Declaration of Helsinki with approval by the Institutional Review Board of Henan Eye Hospital (HNEECKY-2019 (15)). Written informed consent was obtained from the patients and their guardians.

## Results

### Genetic Analysis

**FIGURE 1:**
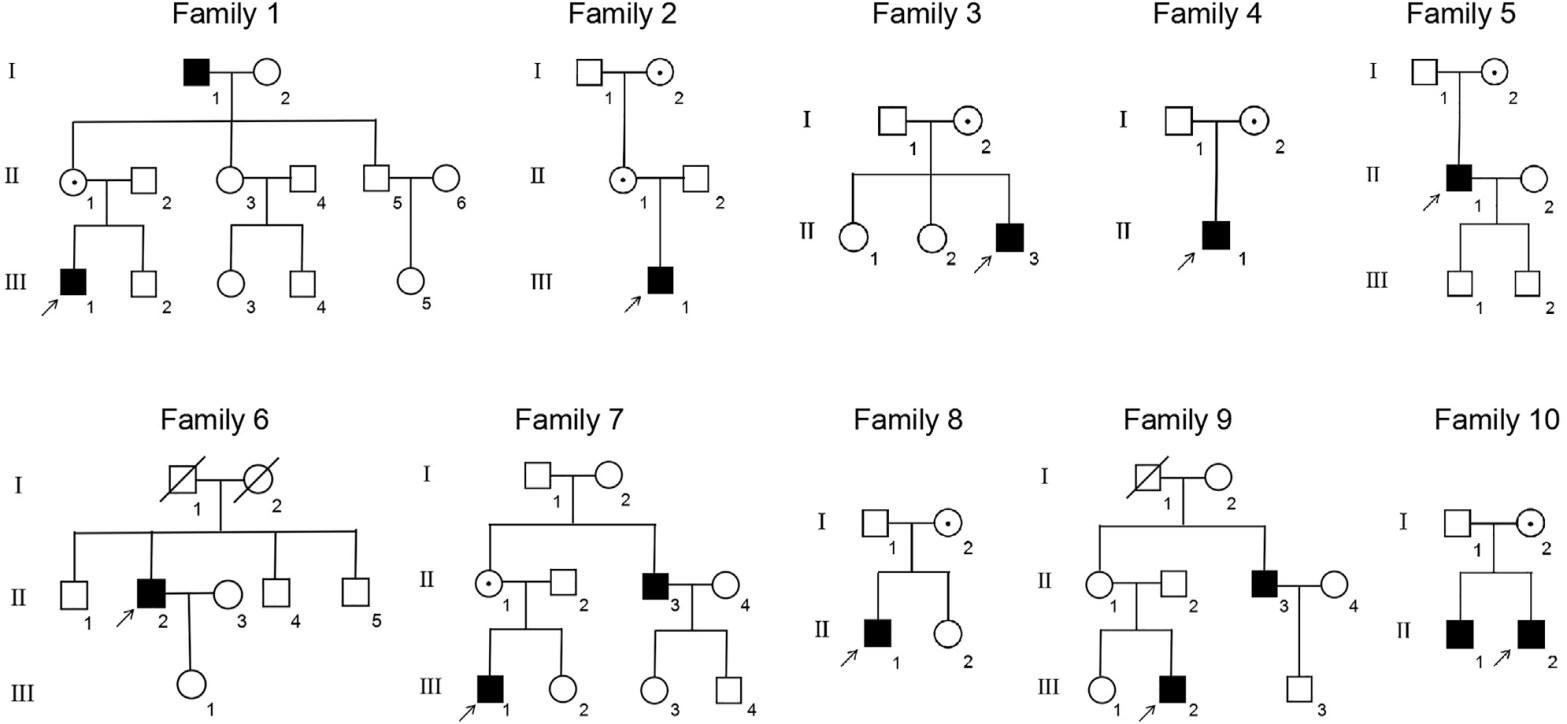
Pedigrees of the XLRS families. ■: male patients; □: normal males; ○: normal females; central dot ○: female asymptomatic carriers; ↑: probands; oblique line □: deceased male; and oblique line ○: deceased female.

**FIGURE 2:**
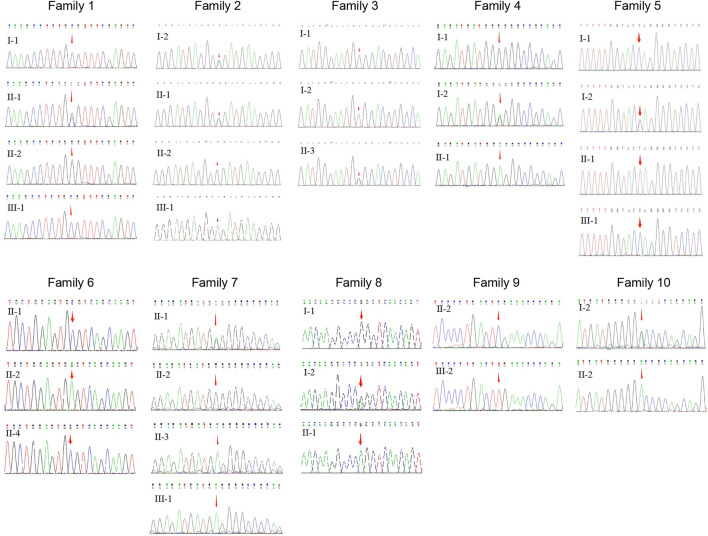
Sequence chromatography of the XLRS families.

This study included 11 male patients from 10 Chinese families (Figure 1; Table 1) with an average age of 19.9 ± 21.6 years. A total of one novel and nine previously reported mutations were detected, including eight missense mutations and two termination mutations. The pathogenicity assessment was carried out according to the ACMG guidelines, and the variations were suspected to be likely pathogenic in 6 (60%) and pathogenic in 4 (40%) mutations. A Sanger sequencing diagram of all families is shown in Figure 2. All of the patients were co-segregated with the disease in their families except patient 10, whose mother’s blood sample was not available.

**TABLE 1:** Genotypes and clinical characteristics of XLRS patients.

Patient no.	Family no.	Age	Mutation	Exon	States	ACMG score	rs ID	PolyPhen-2	SIFT	Mutation Taster	Novel/reported	BCVA (OD/OS)	Foveal Retinoschisis (OD/OS)	Peripheral Retinoschisis (OD/OS)
1	1, III-1	4	c.208G > C (*p*.G70R)	4	Hemi	LP (PM2_Supporting + PP3+PM5+PP1+PS4)	rs62645894	Probably_damaging	Damaging	Disease_causing	Reported	0.2/0.2	+/+	-/-
2	2, III-1	4	c.598C > T (*p*. R200C)	6	Hemi	P (PM2_Supporting + PP3+ PS4)	rs281865357	Probably_damaging	Damaging	Disease_causing	Reported	0.2/0.2	+/+	-/-
3	3, II-3	6	c.626G > A (*p*.R209H)	6	Hemi	LP (PM2_Supporting + PP3+PM1+PS4)	rs281865362	Probably_damaging	Tolerable	Disease_causing	Reported	0.15/0.2	+/+	-/-
4	4, II-1	6	c.577C > T (*p*.P193S)	6	Hemi	LP (PM2_Supporting + PP3+PM1+PS4)	rs281865351	Probably_damaging	Damaging	Disease_causing	Reported	0.5/0.3	+/+	+/+
5	5, II-1	57	c.98G > A (*p*.W33X)	3	Hemi	P (PVS1+PM2_Supporting + PP3)	None	None	None	Disease_causing_automatic	Reported	0.04/0.03	A/A	+/+
6	6, II-2	60	c.657C > A (*p*.C219X)	6	Hemi	P (PVS1+PM2_Supporting + PP3)	None	None	None	Disease_causing	Novel	0.06/0.08	A/A	A/A
7	7, III-1	18	c.578C > T (*p*.P193L)	6	Hemi	LP (PM2_Supporting + PP3+PM1+PS4)	rs281865352	Probably_damaging	Damaging	Disease_causing	Reported	0.05/0.3	+/+	+/+
8	7, II-3	39	c.578C > T (*p*.P193L)	6	Hemi	LP (PM2_Supporting + PP3+PM1+PS4)	rs281865352	Probably_damaging	Damaging	Disease_causing	Reported	0.4/FC	+/+	+/+
9	8, II-1	7	c.305G > A (*p*.R102Q)	4	Hemi	P (PM2_Supporting + PP3 +PS4)	rs61752068	Probably_damaging	Damaging	Disease_causing_automatic	Reported	1.0/0.2	-/-	-/+
10	9, III-2	8	c.214G > A (*p*.E72K)	4	Hemi	LP (PM2_Supporting + PP3 +PS4)	rs104894928	Probably_damaging	Tolerable	Disease_causing	Reported	0.3/0.05	+/+	-/+
11	10, II-2	10	c.574C > T (*p*.P192S)	6	Hemi	LP (PM2_Supporting + PP3+PM1+PS4)	rs61753174	Probably_damaging	Tolerable	Disease_causing	Reported	0.3/HM	+/A	+/+

OS: left eye. OD: right eye. Hemi: hemizygous. LP: likely pathogenic. P: pathogenic. HM: hand move. FC: finger count. +: present. -: absent. A, atrophy.

### Phenotype Analysis

**FIGURE 3:**
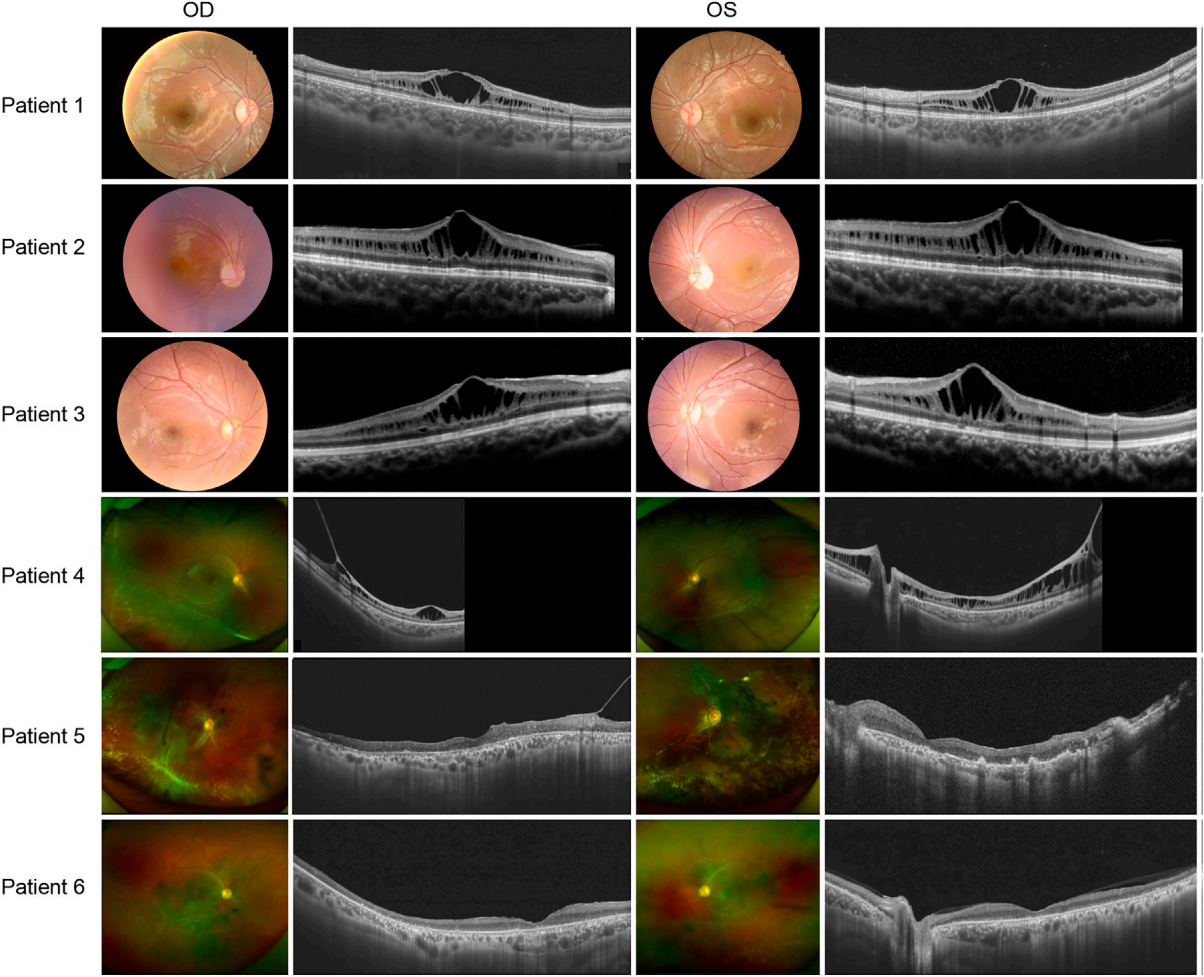
Colored fundus photographs and OCT B scan images of patients 1-6.

There was no significant correlation between the genotype and phenotype in XLRS patients. Patients 1, 2, and 3 presented with typical XLRS appearances, including a poke-like schisis pattern in the fovea bilaterally, without peripheral retinoschisis. Under SS-OCT, the light reflection of the fovea macula ellipsoid zone (EZ) was reduced to various degrees or even disappeared (Figure 3).

The following patients presented with complex fundus appearance: Patient 4 showed extensive interlaminar cleavage in both eyes, and inner retinal layer detachment and bullous peripheral schisis were evident. Two older victims, patients 5 and 6, had poor BCVA, presumably due to extensive retinal atrophy. The fundus of patient 5 showed extensive pigmentation, numerous yellow dendritic-shape lesions, and bullous splitting of the inner retina in the peripheral area (Figure 3).

**FIGURE 4:**
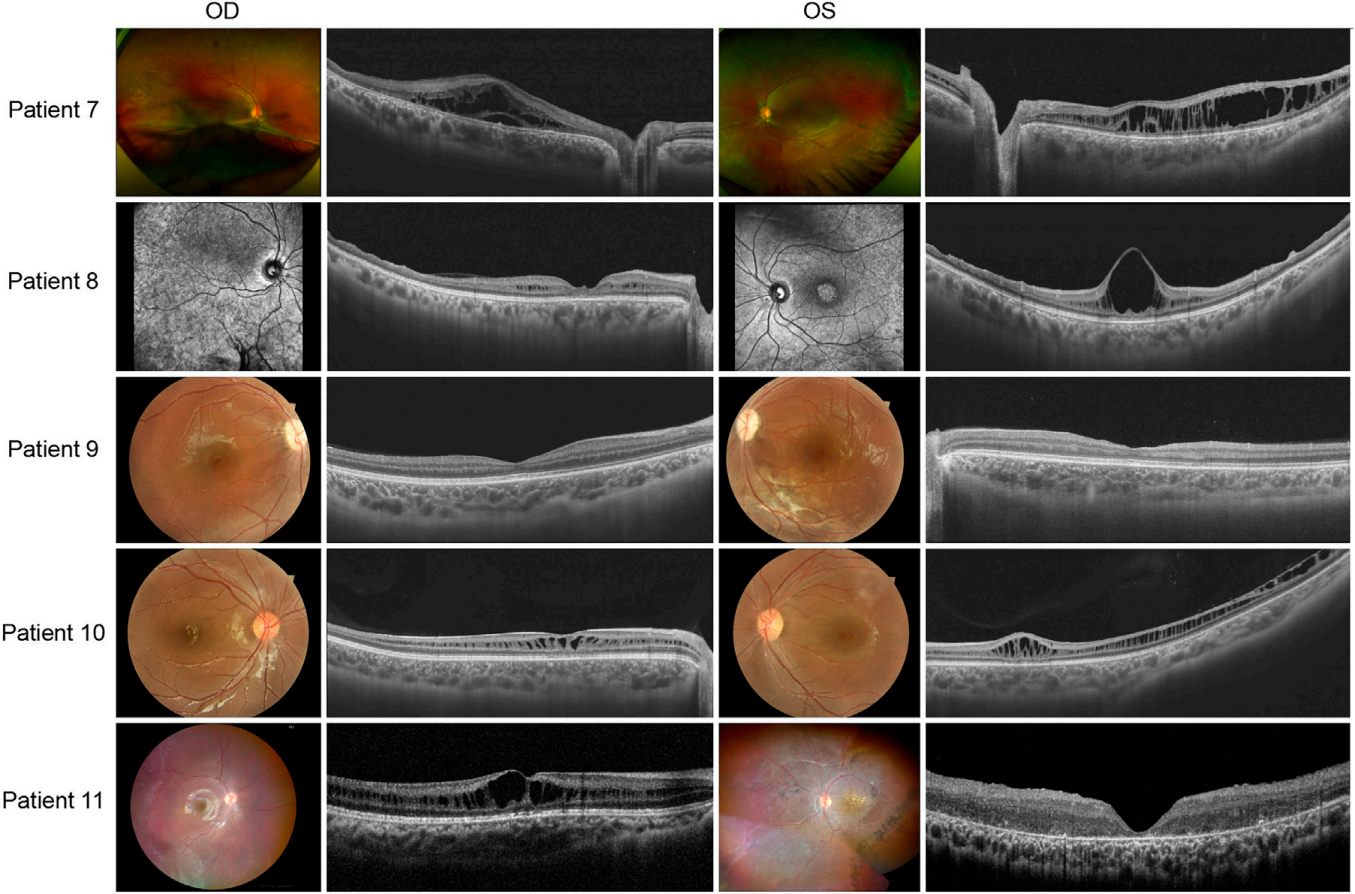
Colored fundus photographs and OCT B scan images of patients 7–11. The fundus images of patient 8 are OCT SLO images.

Patients 7 to 11 presented asymmetric fundus manifestations as well as BCVA. In patient 7, while vitreous veils were seen in both eyes, a severe proliferative membrane could be seen only in his right eye. A large cystic schisis involving the inner nuclear layer (INL), outer plexiform layer (OPL), and ONL was observed. The atrophy of the retina in the outer layers of the right eye was more severe than in the left eye, which could explain the worse vision (Figure 4). Patient 8, the uncle of patient 7, was a middle-aged male. SS-OCT showed peripheral retinas presented with bullous retinoschisis of both eyes (Figure 4). However, uneven elevation of foveal cysts schisis in both eyes was evident, and the right eye had worse vision than the contralateral eye, which might be associated with an early stage of atrophy. In patient 10, varying degrees of macular retinoschisis was seen in both eyes, but peripheral retinoschisis was noticed only in the left eye (Figure 4). Patient 11 had a 13-year-old brother who had also been diagnosed with XLRS. Vitreous veils were present in the 11th patient’s peripheral retinas of both eyes, but the appearance of the macula was substantially different. While the right eye appeared to have a typical cyst cleavage, the left exhibited atrophy of the macula, possibly associated with a previous therapeutic surgery for retinal detachment. The laser photocoagulation spots in the peripheral retina were visible (Figure 4).

**FIGURE 5:**
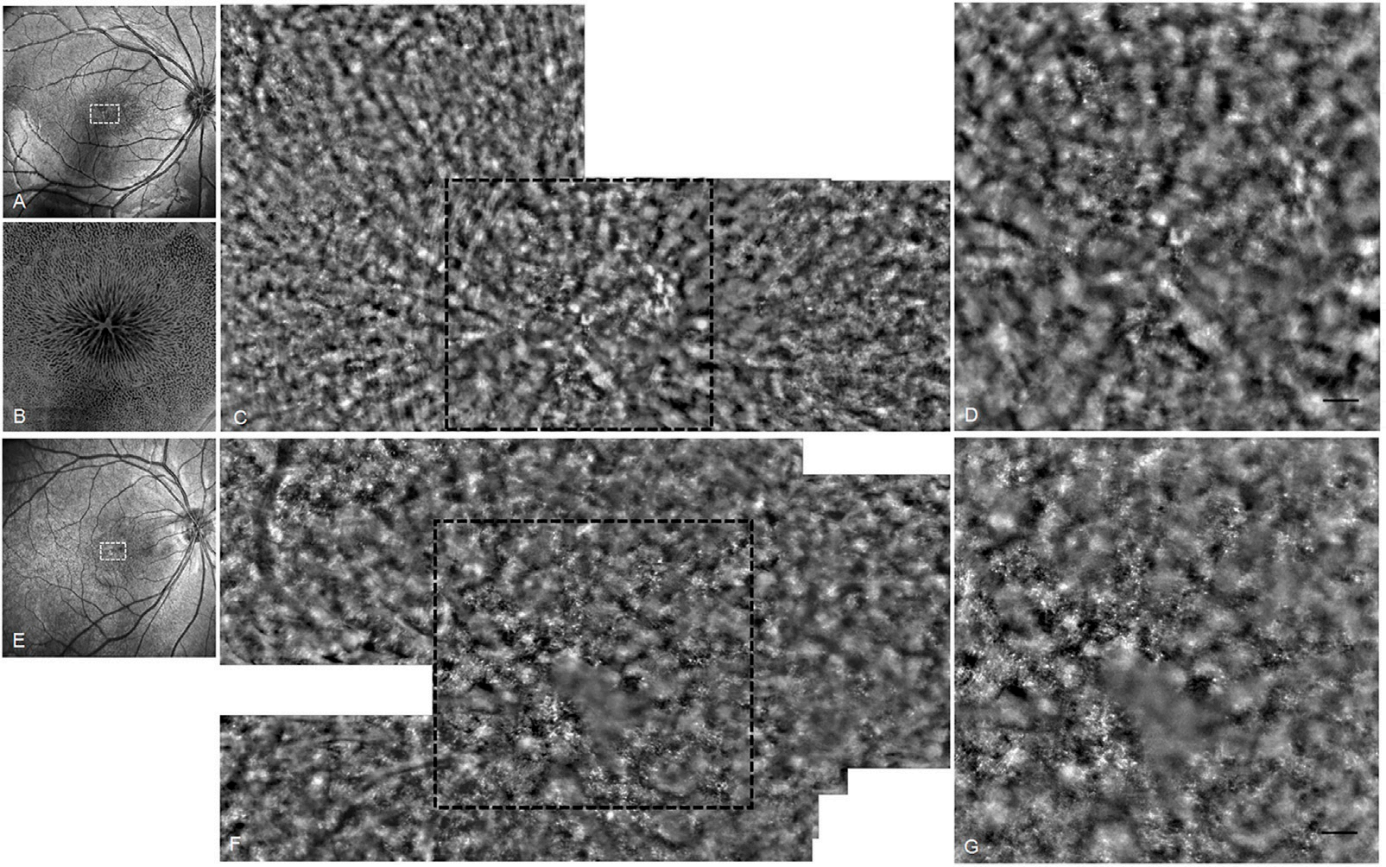
OCT and AO fundus image of patients 10 and 11. **(A)** OCT SLO image of the right eye of patient 10. **(B)** 6 mm × 6 mm OCT *en face* image of right macular of patient 10. **(C)** AO fundus image corresponding to the white dotted box of (A). **(D)** Enlarged black dotted box in **(C)**. The cones appear as small highlighted dots. **(E)** OCT SLO image of the right eye of patient 11. **(F)** AO fundus image corresponding to the white dotted box of **(E)**. **(G)** Enlarged black dotted box in **(F)** (scale bar: 100 μm).

Application of advanced techniques including AO and OCT Angio (OCTA) provided more detailed and valuable information in understanding the phenotype of XLRS. Foveal photoreceptor cells showed accumulation and atrophy in AO images (Figure 5). The density of cones decreased and the cells became more irregular to varying degrees. In *en face* OCTA, the cystic schisis of the INL was shown as a central starlike radial performance, a symbolic sign of the disease.

**FIGURE 6:**
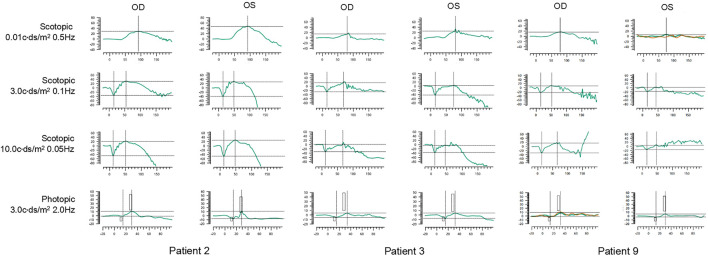
ERG graphs of patients 2, 3, and 9. The first line shows dark-adapted 0.01 ERG. The second line shows dark-adapted 3.0 ERG. The third line shows dark-adapted 10.0 ERG. The fourth line shows light-adapted 3.0 ERG. The abscissa is in milliseconds, and the vertical coordinate is in microvolts.

**FIGURE 7:**
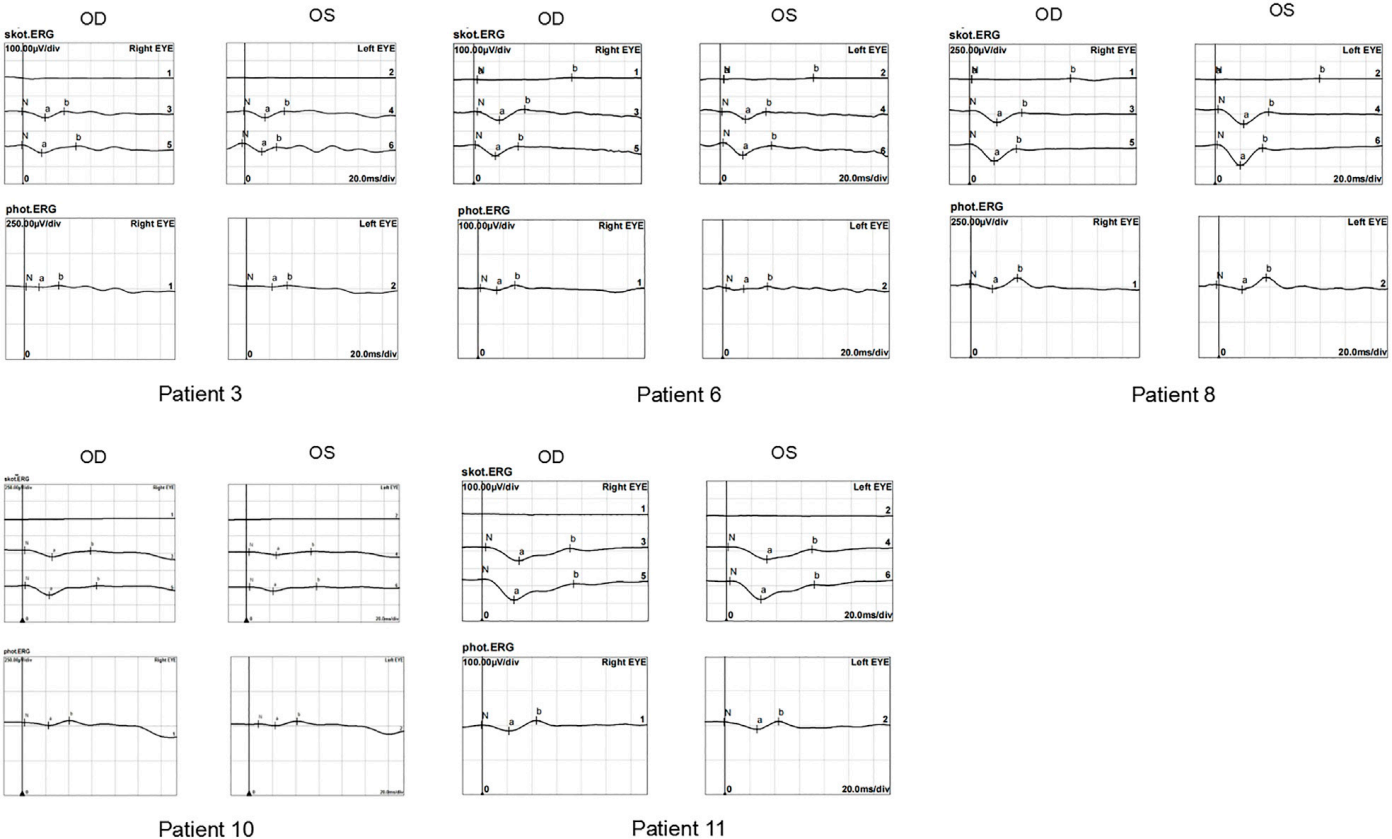
ERG graphs of patients 4, 5, 6, 7, and 10. The top-down waves are, respectively, dark-adapted 0.01 ERG, 3.0 ERG, and 10.0 ERG and light-adapted 3.0 ERG.

The ERG waveforms in these XLRS patients correlated with the severity of the phenotype (Figures 6, 7). In patient 9, who showed asymmetrical fundus changes, dark-adapted ERG revealed a mild decline in rod system function in the nearly normal right eye, but a negative waveform pattern in the peripheral retinoschisis left eye (Figure 6). In patients 5 and 6 with severe macular atrophy, both dark- and light-adapted ERG revealed a severe decline in amplitudes of both eyes (Figure 7).

### BCVA and SS-OCT Parameter Analysis

**FIGURE 8:**
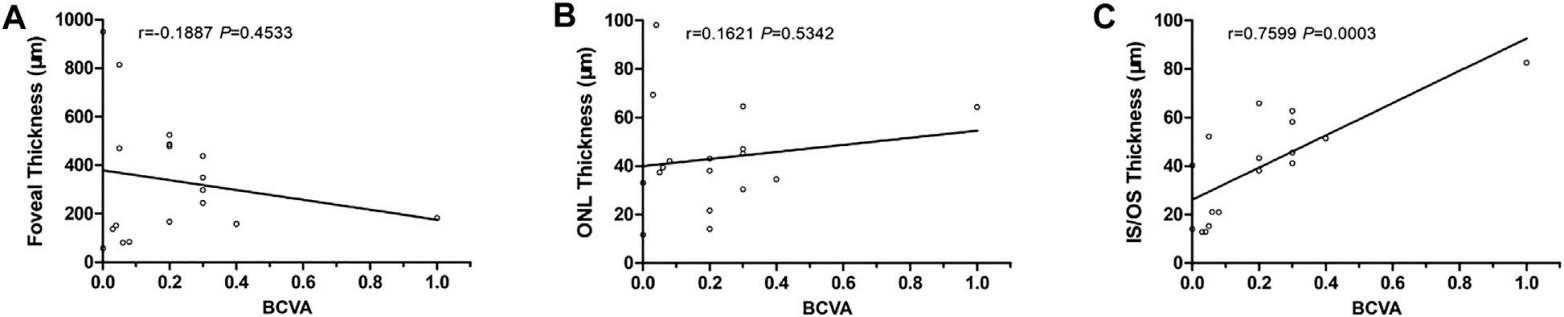
Correlations between BCVA and SS-OCT parameters. **(A)** BCVA vs. foveal thickness. **(B)** BCVA vs. ONL thickness. **(C)** BCVA vs. IS/OS thickness.

The BCVA of group A was 0.32 ± 0.22, and that of group B was 0.04 ± 0.03. Group A was significantly higher than group B (*p* = 0.0019). The thickness of IS/OS in group A was 52.6 ± 14.5 μm, which was significantly higher than in group B, 23.7 ± 14.7 μm (*p* = 0.0007). There were no significant differences in retinal fovea thickness and ONL thickness between the two groups (*p* = 0.9334 and 0.5242). With linear regression, BCVA was significantly associated with the thickness of IS/OS (*p* = 0.0003, r = 0.7599), but not with the thickness of ONL and fovea (Table 2, Figure 8).

**TABLE 2:** BCVA and SS-OCT parameters in XLRS patients. P values less than or equal to 0.05 are shown in bold.

	BCVA	Fovea (μm)	ONL (μm)	IS/OS (μm)
Group A	0.32 ± 0.22	332.8 ± 142.5	40.2 ± 16.4	52.6 ± 14.5
N	14	10	10	10
Group B	0.04 ± 0.03	343.3 ± 358.9	47.2 ± 28.1	23.7 ± 14.7
N	8	8	7	8
*P*	**0.0019**	0.9334	0.5242	**0.0007**

## Discussion

Previous studies have shown no significant association between the *RS1* genotype and XLRS phenotype (Hewitt et al., 2005; Pimenides et al., 2005; Riveiro-Alvarez et al., 2009; Bowles et al., 2011; Vincent et al., 2013; Fahim et al., 2017; Hahn et al., 2021; Vijayasarathy et al., 2021). It was also proved in this study. Although XLRS displays almost full penetrance, its clinical manifestations may be highly variable within the same family (Pimenides et al., 2005; Molday et al., 2012; Kondo et al., 2019). For example, two Chinese XLRS brothers had macular atrophy and retinoschisis, respectively, but there was no change in their BCVA in a 7-year follow-up (Zhang et al., 2021).

The duration of the disease had a significant impact on the clinical appearance. In a study on 56 XLRS patients from 16 British families, macular abnormalities were observed in the eyes of all patients, and foveal schisis was the most common change in patients younger than 40 years (83%). However, a blunted foveal reflex or pigmentary atrophy was more commonly observed in the elderly patients compared with the young victims (85%) (George et al., 1996). Patients with peripheral retinoschisis without concomitant macular schisis or atrophy have rarely been reported (Strupaite et al., 2018; Smith et al., 2020). A large retrospective cohort study of 340 XLRS cases found the phenotype and natural course exhibited a large variability (Hahn et al., 2021). As the disease progressed, the foveal schisis often demonstrated coalescence of the microcysts, forming a large posterior schisis cavity and subsequently a collapse of the foveal schisis cavity, resulting in a blunted foveal reflex in older patients. Patients with bullous peripheral cavities could progress to spontaneous flattening and eventual reattachment, resulting in residual pigmented demarcation lines. Vitreous veils, if present, could fragment over time (Tantri et al., 2004; Rao et al., 2018; Cozzupoli et al., 2020). Patients with onset age less than 1 year had more complications such as retinal detachment and vitreous hemorrhage (Huang et al., 2020).

Generally, XLRS presents a symmetric fundus appearance in both eyes. Asymmetric retinoschisis or macular atrophy cases were rarely reported (Chen et al., 2020). However, it should be noted that various phenotypes may be displayed in the two eyes. The cases in this study exhibited atypical phenotypes, such as the asymmetrical phenotype in patients 8, 9, and 11 as well as the complex phenotype in patients 5 and 6, which made clinical diagnosis very challenging. The mutation c.657C > A (*p*.C219X) carried by patient 6 is a novel nonsense mutation. It is located on the last exon (exon 6) of the *RS1* and may cause premature termination of protein synthesis. It has been reported that exons 4–6 (63–219 amino acids) of the *RS1* gene encode a highly conserved discoidin domain critical to the function of this protein (Wu and Molday, 2003; Wu et al., 2005b). c.655-679del in the C-terminal of this protein will change the Cys219 residue, which is 100% conserved and is the last amino acid of the discoidin domain (Consortium, 1998). Nevertheless, the reasons for the asymmetric and atypical manifestations remained unknown. It might be due to the fact that the two eyes were at different stages of the disease or the genotype was simply different. Several studies have shown that visual acuity decreases gradually with age and progression of macular degeneration (George et al., 1996; Pimenides et al., 2005). On the other hand, Chen et al. suggested that patients carrying null or run-on mutations have more severe visual impairments than those harboring missense mutations, and retinal pigment epithelium (RPE) pigment migration was more frequently observed (Chen et al., 2020; Zhang N. et al., 2021). Other studies have shown that patients with peripheral retinoschisis had worse visual acuity, in which 0.9 percent of the macula appeared normal (Xiao et al., 2021). In our 9th case, although the right eye had better BCVA than the left eye, mild abnormalities had already occurred as shown by ERG. In the 5th and 6th cases, we could not exclude the possibility that patients had typical spoke-like schisis manifestations when they were young, while the examinations applied in the current study were not available at that time. Clearly, further follow-up and comparative analysis are necessary.

There are different views on the causes of visual impairment in XLRS patients. In this study, we found that the residual visual acuity does not appear to be related to the height of the foveal schisis or the continuity of the ellipsoid zone. Nevertheless, BCVA appeared closely related to the structure and function of the outer retina. When the outer layer of the retina, including the ONL, EZ, chimeric zone, and RPE, underwent degeneration, the visual acuity was severely decreased. Ling et al. suggested that poor visual acuity in the early stage of the disease was possibly related to the marked disruption of EZ band and decrease in central foveal thickness over time (Ling et al., 2020). Yang et al. suggested that defects of the cone outer segment tips line and decrease in outer segment lengths, as well as other defects of the photoreceptor microstructure, might be closely related to poor vision in XLRS (Yang et al., 2014). Similarly, Hahn et al. proposed that the integrity of the EZ as well as the outer segment length may be important for choosing candidates for treatment (Hahn et al., 2021). Our data, by showing the close association between the BCVA and SS-OCT measurements, supported the notion that the integrity of the macular outer retinal was crucial for the maintenance of visual acuity. It might be of great significance in determining prognosis and foreseeing the visual outcome of the emerging gene therapy for this disease.

Although most of the area was blocked by the cystic cavities in the inner retina and the signal of cells could not be well presented, we still, by using AO fundus photography, observed a decrease in cone density and regularity in the cystic schisis macular, further evidence that cone photoreceptors are undergoing degeneration during the disease process. Previous studies have shown that, after treatment with acetazolamide, the number of retinal folds in the AO images was reduced (Akeo et al., 2015), and the cone mosaic of XLRS patients could be imaged because the cystic spaces become smaller in size (Tsang and Sharma., 2018). We believe that, with the application of advanced high-resolution technology, the progression of XLRS will be more clearly presented and better understood at the cellular level.

## Conclusion

With complicated clinical manifestations, a considerable portion of XLRS patients may present various phenotypes. It should be noted that asymmetry in fundus appearance in both eyes could lead to misdiagnosis easily. To avoid misdiagnosis and, consequently, mistaken interventions, genetic testing is highly recommended for patients with macular atrophy or cystic degeneration in the mid-peripheral retina, even in patients with only unilateral involvement. In addition, residual cone photoreceptors were critical for the maintenance of useful vision and might be of great significance in predicting the prognosis of the emerging gene therapy for such condition.

## Data Availability

The original contributions presented in the study are included in the article/Supplementary Material, further inquiries can be directed to the corresponding author.
